# Breaking down the wall: a noncoding RNA network regulates pectin remodeling during tomato ripening

**DOI:** 10.1093/plcell/koag230

**Published:** 2026-08-04

**Authors:** Soledad Traubenik

**Affiliations:** Assistant Features Editor, the Plant Cell, American Society of Plant Biologists, United States; Institute of Plant Sciences Paris-Saclay (IPS2), CNRS, INRAE, Université Paris-Cité, Gif-sur-Yvette 91190, France

Over the past decades, noncoding RNAs (ncRNAs) have reshaped our view of gene regulation, emerging as powerful regulators coordinating gene expression programs across every stage of the plant life cycle. While microRNAs (miRNAs) primarily act as post-transcriptional regulators of gene expression (Mencia et al. 2023), long noncoding RNAs (lncRNAs) have been implicated in a wide range of regulatory processes, including chromatin organization, transcriptional control, and RNA metabolism (Lucero et al. 2021; Chorostecki et al. 2023). Traditionally, lncRNAs have been studied as independent regulatory entities. However, accumulating evidence suggests that interactions among different classes of ncRNAs provide additional layers of gene regulation, thereby expanding the complexity of regulatory networks governing plant development and environmental responses. The concept of target mimicry has established that lncRNAs can indirectly regulate gene expression by modulating miRNA activity; in this mechanism, lncRNAs contain miRNA-binding sites that sequester specific miRNAs through imperfect base pairing, preventing them from repressing their target transcripts. The lncRNA INDUCED BY PHOSPHATE STARVATION 1 (IPS1) provided the first demonstration that a lncRNA could function as a molecular decoy, altering the regulatory output of miR399 through an imperfect interaction containing a central bulge that prevents AGO-mediated cleavage (Franco-Zorrilla et al 2007). What initially appeared to be a specialized regulatory mechanism was later found to be more widespread, with the identification of additional target mimics and increasingly complex RNA regulatory circuits across plant species and biological processes (Jabnoune et al 2013; Wang et al 2024). Collectively, these studies transformed our view of target mimicry from a single regulatory mechanism into a broader framework of interconnected RNA regulatory networks.

Using tomato fruit ripening as a developmental model, Zhao and colleagues (2026) have identified a ncRNA regulatory network that controls fruit softening, providing a compelling example of how RNA-mediated endogenous competition can shape a complex developmental program. Their findings expand the original concept of target mimicry into a broader competing endogenous RNA (ceRNA) network, in which lncRNAs, miRNAs, and coding transcripts interact to coordinate gene expression. The authors identified a ceRNA regulatory module that controls fruit softening in tomato, showing that a ripening-induced lncRNA modulates the expression of pectin-remodeling genes (Fig. 1). Mining ripening-associated transcriptomic datasets, the authors identified RiLinc6978, an intergenic lncRNA (lincRNA) whose expression increases during fruit maturation. Increased RiLinc6978 expression accelerated fruit softening, whereas its disruption delayed ripening, establishing this lincRNA as a regulator of pectin remodeling and cell wall dynamics. But how does a lncRNA influence pectin metabolism? This function was mediated by its interaction with miR9474-5p, whose abundance was negatively correlated with that of RiLinc6978. The authors demonstrated that RiLinc6978 and miR9474-5p co-localize in the nucleus and cytoplasm and physically interact through 2 predicted miRNA response elements. Transient expression assays showed that increasing RiLinc6978 levels reduced miR9474-5p activity, whereas disruption of the lncRNA increased miR9474-5p accumulation, consistent with target mimicry. Functional analyses further revealed that miR9474-5p acts as a negative regulator of fruit softening. Transcriptomic analyses and target validation linked miR9474-5p activity to genes involved in pectin degradation, including pectin methylesterases and other cell wall-modifying enzymes, supporting a model in which RiLinc6978 promotes fruit softening by limiting miR9474-5p-mediated repression of pectin-remodeling genes.

**Figure 1:**
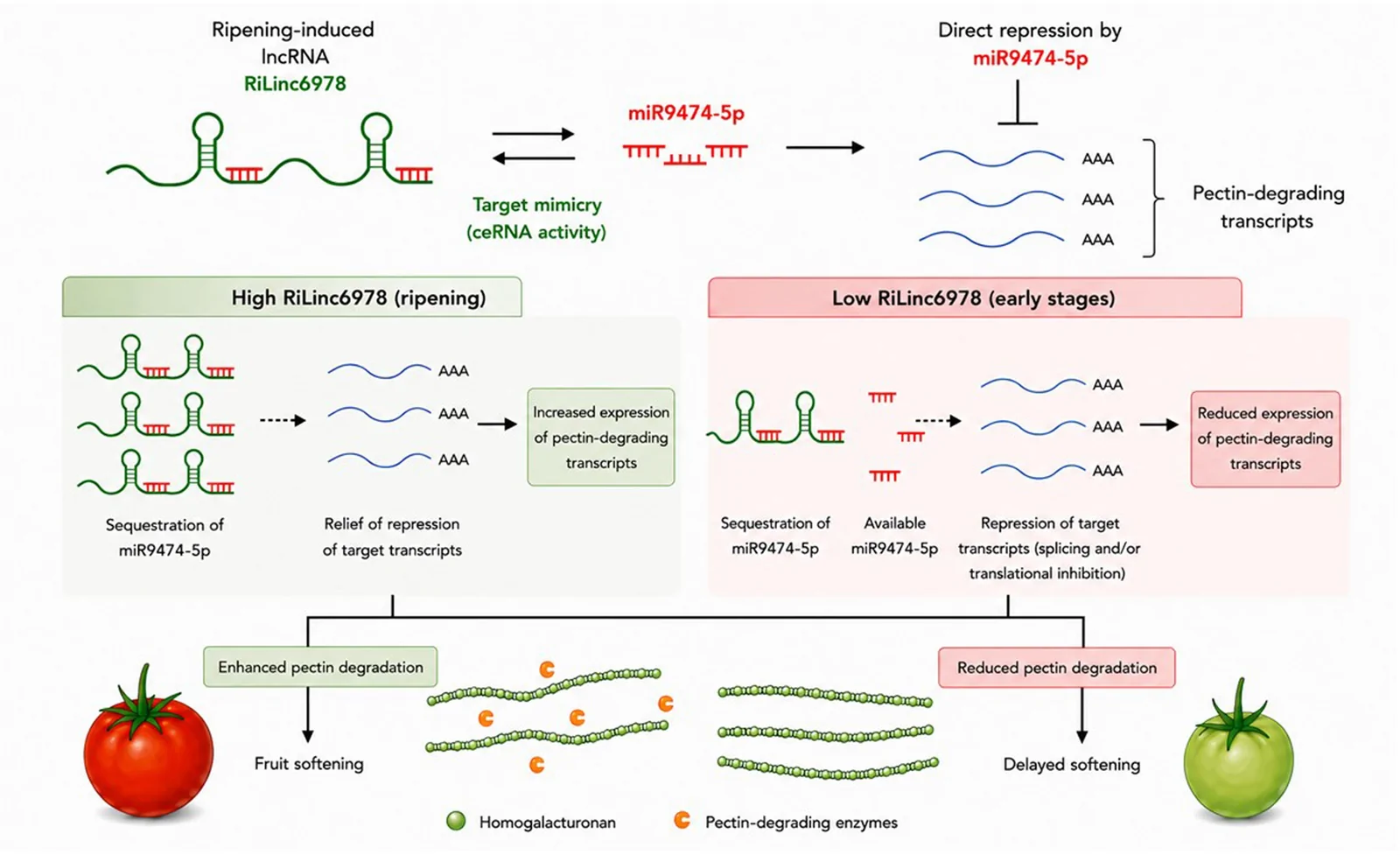
A ceRNA network controls tomato fruit softening through RNA-mediated competition. The ripening-induced lncRNA RiLinc6978 functions as a molecular decoy for miR9474-5p, reducing its regulatory activity toward transcripts involved in pectin degradation. During ripening, increased RiLinc6978 levels sequester miR9474-5p, promoting the expression of pectin-degrading transcripts, enhanced pectin breakdown, and fruit softening. At early developmental stages, lower RiLinc6978 abundance allows miR9474-5p to repress its targets, limiting pectin degradation and delaying fruit softening.

More than identifying a new regulator of fruit softening, Zhao and colleagues expand the growing repertoire of ncRNA-mediated regulatory interactions in plants. This work reinforces the idea that noncoding RNAs are not merely individual regulators acting in parallel, but components of interconnected regulatory networks capable of shaping complex developmental transitions. It also illustrates how target mimicry can be integrated into broader ceRNA networks, where regulatory RNAs influence one another to shape gene expression outputs and developmental outcomes. As additional examples emerge, it is becoming increasingly clear that noncoding RNAs are not simply individual regulators acting in parallel, but components of interconnected regulatory circuits that add an additional layer of control to plant developmental programs.

## Recent related articles in *The Plant Cell*

- (Liu et al., 2025) This study identifies a circular RNA that suppresses rice resistance to bacterial blight by acting as a miR398b sponge and modulating defense-associated reactive oxygen species accumulation.
- (Meena et al., 2024) This study reveals that antisense long noncoding RNAs positively regulate cold-responsive transcription factors, contributing to freezing tolerance in Arabidopsis.
- (Kornienko et al., 2024) This study uncovers pervasive natural variation in Arabidopsis lncRNA expression, linking lncRNA silencing to transposable element-associated chromatin regulation.

## Data Availability

No datasets were generated or analysed.
